# Poorly differentiated mesenteric carcinoma of unknown primary site detected by abscess formation: case report

**DOI:** 10.1186/1477-7819-12-4

**Published:** 2014-01-08

**Authors:** Yukinori Yamagata, Yukari Ando, Keisuke Matsusaka, Hisako Karube, Haruna Onoyama, Susumu Aikou, Hiroharu Yamashita, Kazuhiko Mori, Sachiyo Nomura, Masashi Fukayama, Yasuyuki Seto

**Affiliations:** 1 Department of Gastrointestinal Surgery, the University of Tokyo Hospital, 7-3-1 Hongo, Bunkyo-ku, Tokyo 113-8655, Japan; 2 Department of Pathology, the University of Tokyo Hospital, 7-3-1 Hongo, Bunkyo-ku, Tokyo 113-8655, Japan

**Keywords:** Carcinoma of unknown primary site, Abscess, Mesentery

## Abstract

**Background:**

Carcinoma of unknown primary site (CUP) is said to account for approximately 3 to 5% of all carcinomas. However, an isolated lesion in the abdominal cavity is rare, and there are no reports describing associated abscess formation.

**Case presentation:**

A 76-year-old woman had consulted a previous physician complaining of fever and right lower quadrant abdominal pain. Enhanced computed tomography (CT) showed an abscess formation around the cecum. She was treated conservatively with antibiotics, but the symptoms relapsed and she consulted our hospital. Enhanced CT showed a persistent abscess, a tumorous lesion in the mesentery and right hydronephrosis. Because malignancy could not be ruled out, surgical treatment was selected. At laparotomy, encapsulated abscesses were found on the mesenteric side and outside of the ileocecal region. When we raised the ileocecal region, a tumor was found to be fixed to the right ureter, and there was leakage of white, solid tumor content. This tumor content was submitted to intraoperative frozen section diagnosis which revealed a carcinoma. Ileocecal resection with D3 lymph node dissection and retroperitoneal tumor resection was thus performed. There were no abnormal findings in the uterus and adnexa, nor any evidence of peritoneal dissemination. We regarded this case as an incomplete resection and chemotherapy with paclitaxel and carboplatin was administered. The patient has remained alive and disease-free for almost one year since the primary operation.

**Conclusion:**

We described a case with mesenteric CUP discovered during surgery for an intra-abdominal abscess. It is necessary to pay attention to treatment-resistant intraperitoneal abscesses as they may accompany a tumor.

## Background

Carcinoma of unknown primary site (CUP) is defined as the presence of metastatic cancer in the absence of an identifiable primary tumor site, even after thorough clinical examination and diagnostic studies. CUP accounts for approximately 3 to 5% of all malignancies, and is thus not particularly rare [1,2]. However, CUPs are a heterogeneous group of malignant neoplasms with different biological characteristics. In CUP cases, finding an isolated lesion in the abdominal cavity is rare, and associated abscess formation is extremely rare. Herein, we report a CUP case in which a tumor was discovered during surgery for a treatment-resistant intra-abdominal abscess.

## Case presentation

The patient was a 76-year-old woman who had a history of surgery for papillary thyroid cancer and cesarean section with uterus bicornis.

**Figure 1:**
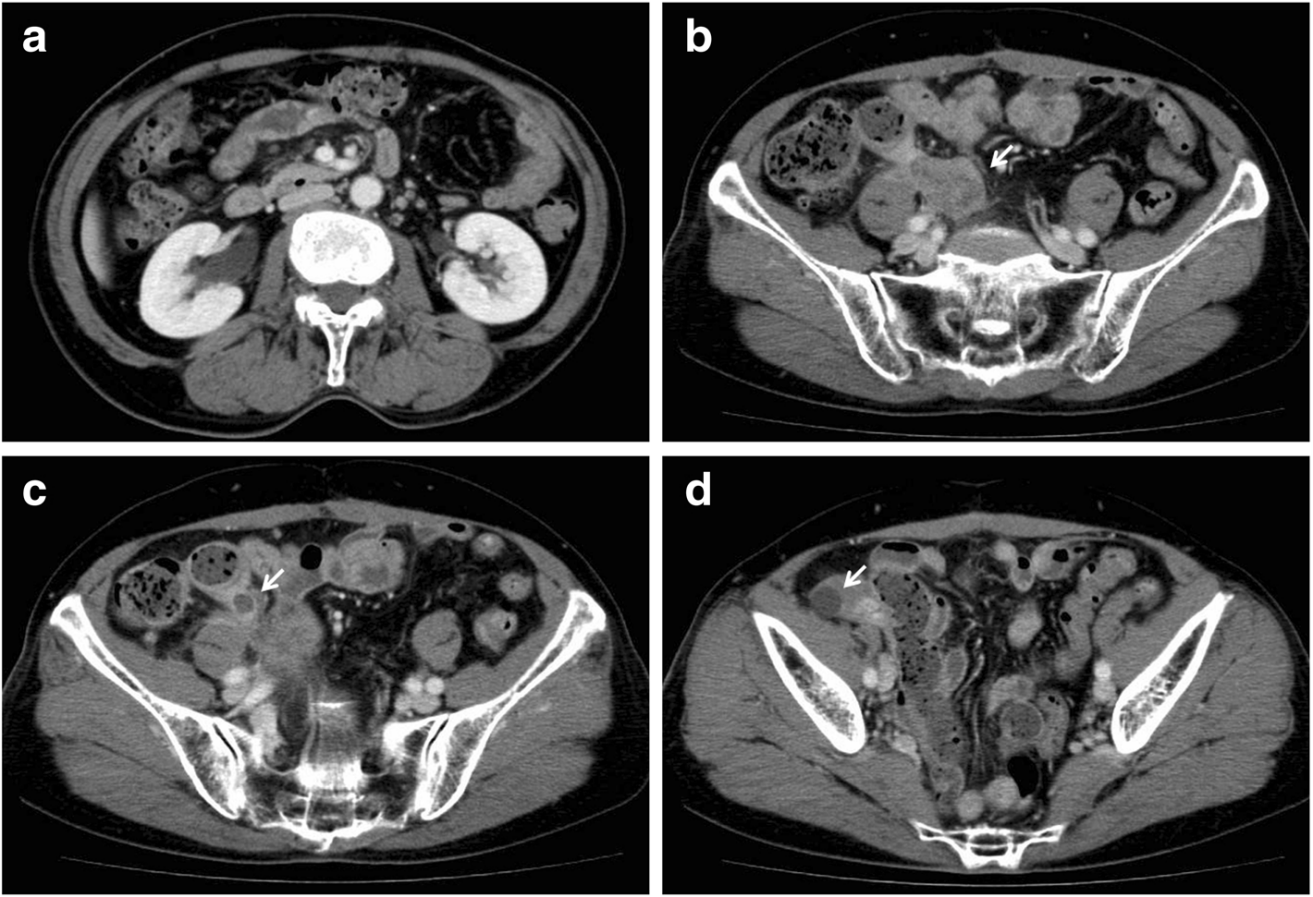
**Re-examination by enhanced computed tomography (CT) scan.** The CT scan shows right hydronephrosis **(a)**, a tumorous lesion in the mesentery **(b)**, and persistent encapsulated abscesses on the mesenteric side **(c)** and on the outside **(d)** of the ileocecal region.

She had consulted a previous physician complaining of fever and right lower quadrant abdominal pain. A marked inflammatory reaction was observed and enhanced computed tomography (CT) showed abscess formation around the cecum. An intra-abdominal abscess was diagnosed and the patient was treated conservatively with antibiotics, but her symptoms relapsed after discharge and she was referred to our hospital. The inflammatory reaction showed exacerbation and cancer antigen 125 (CA125) was mildly elevated (78 U/mL). Enhanced CT was performed again and showed a persistent abscess, a tumorous lesion in the mesentery and right hydronephrosis (Figure 1). Because malignancy could not be ruled out, surgical treatment was selected.

**Figure 2:**
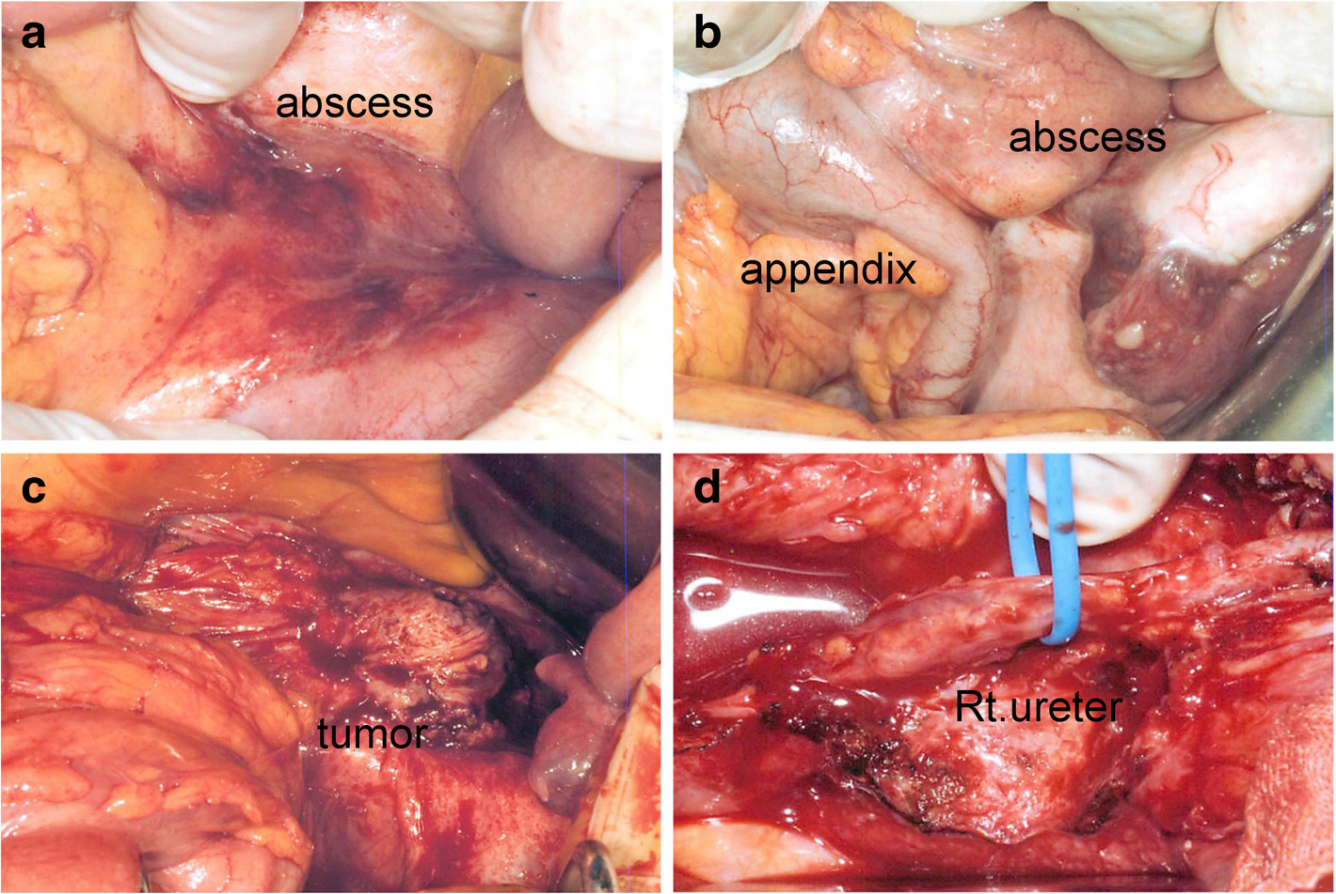
**Intraoperative findings.** Encapsulated abscesses can be seen on the mesenteric side **(a)** and outside of the ileocecal region **(b)**. When we raised the ileocecal region, a tumor was found to be fixed to the right ureter **(c)**. A ureteral stent was inserted into the right ureter; the tumor was then separated and removed from the ureter **(d)**.

At laparotomy, there were no ascites but encapsulated abscesses were present on the mesenteric side and outside of the ileocecal region (Figure 2a,b). When we raised the ileocecal region, a tumor was found to be fixed to the right ureter and there was leakage of white, solid tumor content (Figure 2c). This tumor content was submitted to intraoperative frozen section diagnosis, which demonstrated a ‘carcinoma’. Also, enlarged lymph nodes were observed along the ileocolic artery. For the above reasons, we performed ileocecal resection with D3 lymph node Dissection. A ureteral stent was then inserted into the right ureter, and the tumor was separated and removed from the ureter with scissors (Figure 2d). The ureteral stent was easily inserted without resistance and urine cytology was negative for carcinoma. After tumor removal, we searched the abdominal cavity thoroughly, but there were no abnormalities of the uterus and adnexa, nor any evidence of peritoneal dissemination.

**Figure 3:**
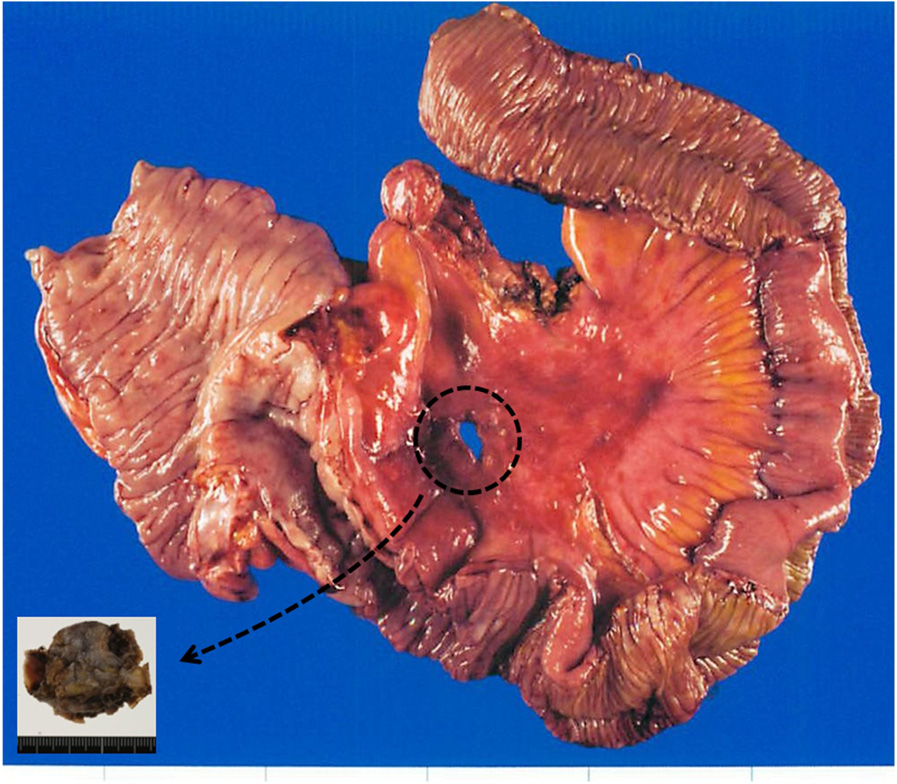
**Macroscopic findings of the tumor and ileocecal lesion.** No changes involving the mucosal surface of the cecum or the terminal ileum were detected.

**Figure 4:**
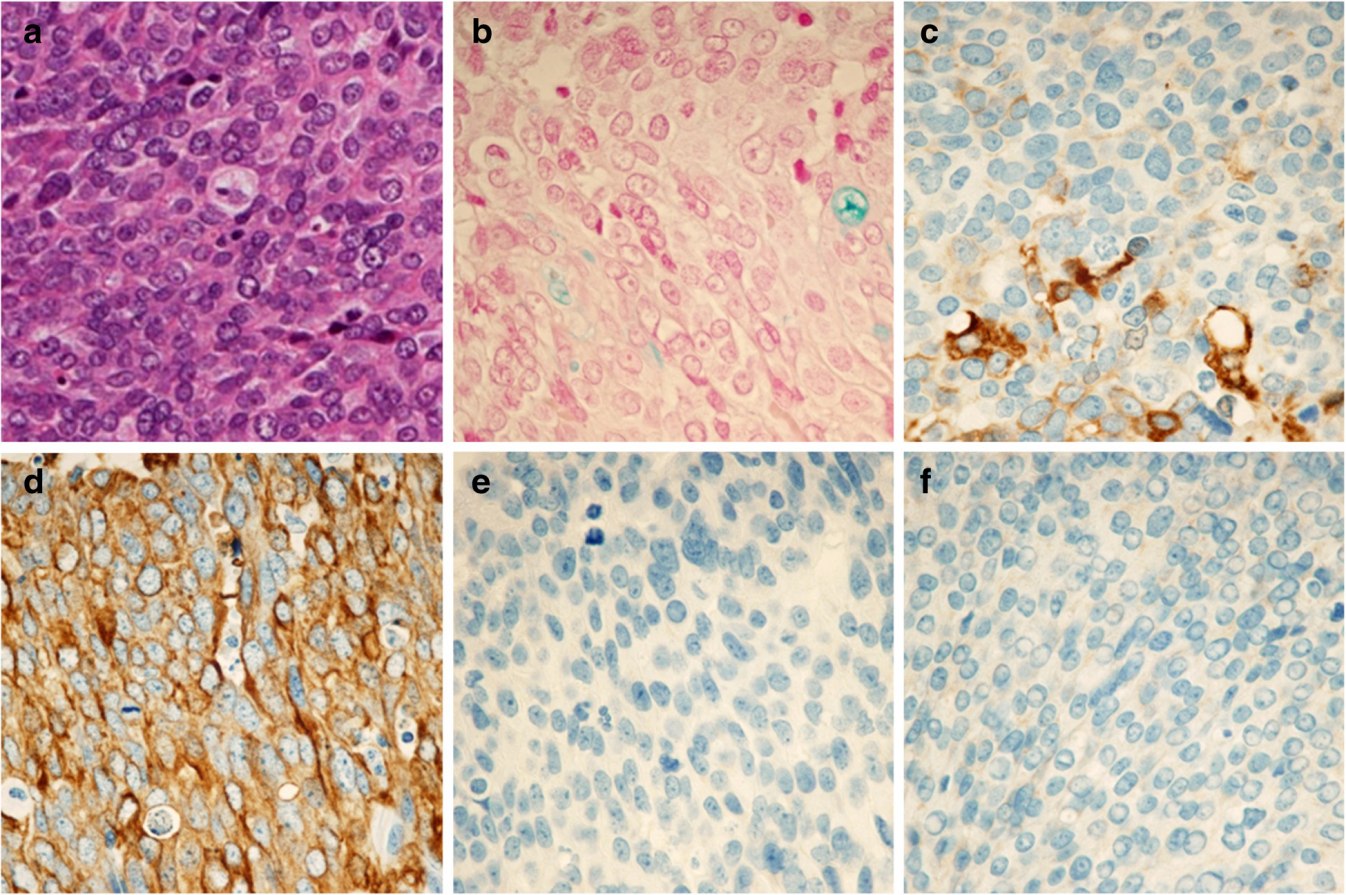
**Histological and immunohistochemical findings of the tumor.** Hematoxylin-eosin (HE) staining reveals poorly differentiated carcinoma **(a)**. Alcian blue (AB) staining showed moderate production of mucin accompanied by a partial ductal structure **(b)**. Immunohistochemistry was partially positive for CK34βE12 (about 20% of tumor) **(c)**, positive for AE1/AE3 **(d)**, negative for CDX2 **(e)**, and negative for TTF-1 **(f)**.

The pathological diagnosis was poorly differentiated carcinoma, but there were no changes involving the mucosal surface of the cecum or the terminal ileum (Figure 3). The mesenteric lymph nodes were free of metastasis. Hematoxylin-eosin (HE) staining showed findings of poorly differentiated carcinoma, and alcian blue (AB) staining showed moderate production of mucin accompanied by a partial ductal structure. Immunohistochemistry showed the tumor to be positive for AE1/AE3 (pancytokeratin), cytokeratin(CK)7, CK34βE12 (about 20% of tumor) and Wilms tumor gene 1 (WT-1), with nonspecific reactions for CK5/6, p63, carcinoembryonic antigen (CEA), estrogen receptor (ER) and vimentin, while CK20, thyroid transcription factor 1 (TTF-1), caudal type homeobox 2 (CDX2), gross cystic disease fluid protein-15 (GCDFP-15), calretinin, D2-40 and CD10 were negative (Figure 4). No pathogen was detected in bacterial culture of the pus. After surgery, esophagogastroduodenoscopy and colonoscopy were conducted, and no gastrointestinal tract lesions were observed. Gynecologic and transvaginal ultrasonography (US) examinations revealed no abnormal findings. Positron emission tomography (PET) showed no abnormal uptake.

Because the tumor content had leaked into the abdominal cavity, we regarded this case as having undergone incomplete resection and six courses of chemotherapy with paclitaxel (PTX, 220 mg/body at day one) and carboplatin (CBDCA, 360 mg/body at day one) were administered at intervals of four weeks. The patient has remained well and disease-free for almost one year, to date, since the primary operation.

## Discussion

CUP accounts for approximately 3 to 5% of all malignancies. Finding CUP as an isolated lesion in the abdominal cavity is rare and there are no reports, to our knowledge, of cases with abscess formation. This is thus the first report of CUP discovered during surgery for an intraperitoneal abscess.

Histopathological examination, especially the immunohistochemical approach, is important for identifying the primary lesion in patients with CUP [1]. In our present case, AE1/AE3 was positive; AB staining showed moderate production of mucin accompanied by a partial ductal structure; and squamous cell markers CK34βE12, CK5/6 and p63, were also slightly positive. However, HE staining showed findings of carcinoma with low differentiation overall, indicating a final diagnosis of ‘poorly differentiated carcinoma’.

Efforts were made to identify the primary tumor. The gastrointestinal tract was negative because CDX-2 was negative. The patient had a past history of papillary thyroid cancer but TTF-1 was negative and the nuclear findings were also inconsistent with thyroid cancer recurrence. Furthermore, the possibility of primary ovarian cancer was considered based on the CK7 positive, CK20 negative, calretinin negative, WT-1 positive, CEA nonspecific and ER nonspecific findings. However, intraoperative findings and postoperative gynecological examination, transvaginal US, and PET revealed no abnormalities and there were no findings indicating a primary ovarian tumor.

CUPs are a heterogeneous group of malignant neoplasms with different biological characteristics. Our patient’s age and pelvic tumor with an increased CA125 value suggested so-called ‘serous papillary peritoneal carcinomatosis’ [3]. However, findings indicating that the tumor ‘was not adenocarcinoma’, ‘was confined to the retroperitoneal cavity with no exposure to the abdominal cavity’ and ‘was not accompanied by peritoneal dissemination’ were also noted in the pathological report. These observations do not meet the criteria for serous papillary peritoneal carcinomatosis.

In addition, abscess formation was observed in this case. The tumor was in contact with the gastrointestinal tract, but did not penetrate the digestive tract. Culture of the pus yielded no infectious organisms. For these reasons, it was suggested that this tumor was not gastrointestinal in origin and that the abscesses had formed due to necrosis of the tumor. From the location of the abscesses, cecal diverticulitis had been suspected by the previous physician, and conservative treatment with antibiotics had been selected. However, the abscesses were unaffected by this treatment. When she consulted our department, re-examination by CT raised suspicion of a tumorous lesion and surgical management was chosen, finally providing a definitive diagnosis. It is necessary to pay attention to treatment-resistant intraperitoneal abscesses as they may accompany a tumor.

With CUP, as its name suggests, a ‘metastatic’ carcinoma with an unknown primary site, the prognosis is generally poor and chemotherapy has been the basis of treatment. Platinum-based or platinum-taxane combination chemotherapy is common [4-6]. Depending on the characteristics and the location of the tumor, multimodal therapy combining radiation, cytoreductive surgery and chemotherapy is sometimes performed. In this case, because abscesses were also present and the tumor was confined to the retroperitoneum, we performed surgery followed by PTX and CBDCA combined chemotherapy. The patient has remained recurrence free for one year, to date, since the initial operation.

## Conclusion

We have presented a case with mesenteric poorly differentiated carcinoma of unknown primary site with abscess formation. It is necessary to pay attention to treatment-resistant intraperitoneal abscesses because of their possible association with malignant tumors.

## Consent

Written informed consent was obtained from the patient for publication of this case report and accompanying images. A copy of the written consent is available for review by the Editor-in-Chief of this journal.

## Abbreviations

AB: Alcian blue; CA125: Cancer antigen 125; CBDCA: Carboplatin; CDX2: Caudal type homeobox 2; CEA: Carcinoembryonic antigen; CK: Cytokeratin; CT: Computed tomography; CUP: Carcinoma of unknown primary site; ER: Estrogen receptor; GCDFP-15: Gross cystic disease fluid protein-15; HE: Hematoxylin-eosin; PTX: Paclitaxel; TTF-1: Thyroid transcription factor 1; US: Ultrasonography; PET: Positron emission tomography; WT-1: Wilms tumor gene 1.

## Competing interests

The authors declare that they have no competing interests.

## Authors’ contributions

All authors have been involved in the management of the patient and in the conception of the manuscript. YY, KM and YS have been involved in drafting the manuscript or revising it critically for important intellectual content. All authors read and approved the final manuscript.
